# Scale Space Methods for Analysis of Type 2 Diabetes Patients' Blood Glucose Values

**DOI:** 10.1155/2011/672039

**Published:** 2011-02-22

**Authors:** Stein Olav Skrøvseth, Fred Godtliebsen

**Affiliations:** ^1^Norwegian Centre for Integrated Care and Telemedicine, University Hospital of North Norway, 9038 Tromsø, Norway; ^2^Department of Mathematics and Statistics, University of Tromsø, 9037 Tromsø, Norway

## Abstract

We describe how scale space methods can be used for quantitative analysis of blood glucose concentrations from type 2 diabetes patients. Blood glucose values were recorded voluntarily by the patients over one full year as part of a self-management process, where the time and frequency of the recordings are decided by the patients. This makes a unique dataset in its extent, though with a large variation in reliability of the recordings. Scale space and frequency space techniques are suited to reveal important features of unevenly sampled data, and useful for identifying medically relevant features for use both by patients as part of their self-management process, and provide useful information for physicians.

## 1. Introduction

Diabetes type 2 has emerged as a major health concern in the western world over the last decade, where lifestyle and diet are considered the most important factors for the incidence rise. Type 2 diabetes is a complex disease characterized by both genetic and environmental factors. Diabetes affects some 220 million persons worldwide where approximately 90% of the cases are type 2 diabetes [1]. Since these patients require considerable medical attention, they constitute a significant cost to society, and much effort is done to provide them with tools that can help patients administer and monitor their disease and encourage a change in lifestyle. As such, there exists a multitude of self-help tools which aim to empower the patients.

One such tool is a mobile phone-based application with an integrated sensor network that has been developed at the Norwegian Centre for Integrated Care and Telemedicine called the * Few Touch* application [2, 3]. The software constituting the user interface is running on the mobile phone, enables wireless and automatic recordings of step count data and blood glucose data, in addition to functionality for users to input dietary information. The aim of this tool is to help the patients in the diabetes self management process, and all data input is done voluntarily. Recorded information can therefore be sporadic, but this approach is shown to yield a high degree of participation over a long period of time, and as such the resulting data set is unique in terms of the extent of the recording period. Patients typically measure their blood glucose concentration (BGC) approximately once per day as part of the self management process, and under the *Few Touch* application these values are automatically transferred to the mobile phone via a Bluetooth adapter at the time of measurement. In this paper we will exclusively focus on the BGC values and not consider values reported by step counters or the dietary registration system. For a variety of technical and personal reasons, some patients did not record BGC for the complete period, and we will only consider in more detail those that record their BGC reliably.

Scale space methods have emerged over the last decade as a set of statistical techniques for exploring features in both one- and two-dimensional data on a variety of scales, in both time and frequency space [4–6]. The fundamental question asked is, in a complicated signal, which features are “really there” as opposed to features that are simply artifacts or “noise.” In our case, the BGC values are not true noise since every data point reflects a true BGC with a negligible error, but outlier recordings on short time scales carry no explanatory power, and we are bound to search for features that emerge on some scale larger than the typical interval. For a time series or density estimate, one can attempt to smooth with a range of bandwidths, and for each bandwidth compute significance intervals to test for significant derivatives or curvatures. Arguably the most common such approach is known as SiZer (Significant Zero-crossing of derivatives) [4], from which several similar tools have been developed. The usefulness of these tools is substantial, though remains largely unknown outside the statistical society.

Scale space methodology has also been applied in frequency space and within a Bayesian framework, though only with evenly sampled data sets [6]. Since the BGC values are very unevenly sampled, even for the “best” patients, this technique is not presently available for these data.

We have applied the SiZer methodology to a dataset collected from 12 patients using the * Few Touch* application over the course of one full year. The data being sampled very unevenly still make them well suited for the SiZer methodology, and allows us to explore periodicities on frequencies larger than the Nyquist frequency by least squares fits to sinusoids.

The full data set consists of twelve patients recorded over the total period from 16 September 2008 through 25 November 2009, a total of 435 days. No patient has recorded BGC every day, and some have recorded for a shorter period than the full year. One patient recorded BGC at least once per day for 373 consecutive days, while four had a longest consecutive period of less than two weeks.

The paper is organized as follows; in Section 2 we will briefly explain the SiZer methodology which is the framework within which the further analysis proceeds. In Section 3 we explore features that emerge when treating the complete dataset, while in Section 4 we break down the analysis into individual patients.  Section 5 contains analysis in frequency space, and we conclude in Section 6.

## 2. The SiZer Methodology

We include a brief overview of the SiZer scheme in this section, a more comprehensive treatment can be found in [4, 7], while some extensions and theoretical justifications exists [5, 6], and several extensions such as a Bayesian version for scatter plots in two dimensions known as B-SiZer [8] have been developed within this framework. When investigating a histogram of values, the canonical way is to smooth with a Gaussian kernel such that important features can be extracted [9]. However, this approach is heavily dependent on the choice of bandwidth, and it is difficult to know which features are significant or not. A number of well-founded techniques exist to specify one optimal data-driven bandwidth, such as the Least-Squares Cross-Validation (LSCV) algorithm [10] or the Sheather-Jones algorithm [11]. The latter, which we have used to highlight some values, attempts to minimize the mean integrated square error by a direct plug-in mechanism. For details, see, for example, [9].

However, it is true that significant structure may emerge at a variety of scales and that the significant features may disappear again at different scales. This was early realized in image processing, where the fact that vision is an inherently multiscale process has been used to develop applications of computer vision [12]. Later, the stringent statistical framework known as SiZer was developed, in which every relevant scale is investigated for significant gradient (or curvature), and the results are presented in a typical SiZer plot, which is a scale space representation of the data where significant changes at a given combination of scale and position, or time, are indicated with different colors. Thus a quick assessment of the data is possible, even in vastly complicated data set, and the important features can immediately be identified and investigated further. Thus one can quickly find important features of the data set, and one can use these as a tool for hypothesis generation.

We treat a scale space in time, where each location is denoted by a time *t* and a scale or bandwidth *h*. Considering values recorded at times *t*_*i*_, *i* = 1,2,…, *N*, we have a smoothed density estimator [9]

(1) $${\hat{f}}_{h}\left(t\right)=\frac{1}{N}\overset{N}{\underset{i=1}{\sum }}K_{h}\left(t-t_{i}\right),$$

where *K*_*h*_(*t*) is some kernel density such as the Gaussian kernel,

(2) $$K_{h}\left(t\right)=\frac{1}{h\sqrt{2\pi }}\exp\;\;\left[-\frac{1}{2}{\left(\frac{t}{h}\right)}^{2}\right].$$

Other kernels can be considered, though it is not suspected that these will reveal much new information. Indeed, the Gaussian kernel has become a de facto standard for density smoothes in part due to the fact that the Gaussian kernel is unique in that it has a monotone decrease of zero crossings of the derivative smooths with increasing bandwidth [13, 14]. This means that features are monotone in scale space. For these reasons, we use the Gaussian kernel exclusively in the following.

The search for the “true” underlying curve *f*(*t*) or its derivative *f*′(*t*) has been all but discarded in the literature, since the estimator ${\hat{f}}_{h}^{\prime }\left(t\right)$ for *f*′(*t*) is biased. Hence, we rather compute the scale space version $f_{h}^{\prime }\left(t\right)=\text{E}\left[{\hat{f}}_{h}^{\prime }\left(t\right)\right]$, where ${\hat{f}}_{h}^{\prime }\left(t\right)$ is an unbiased estimator for *f*_*h*_′(*t*) at each time *t* and scale *h*. Hence, we can assume

(3) $$\frac{{\hat{f}}_{h}^{\prime }\left(t\right)-f_{h}^{\prime }\left(t\right)}{\hat{\text{SD}\left[{\hat{f}}_{h}^{\prime }\left(t\right)\right]}}\sim 𝒩\left(0,1\right)$$

and investigate all values of *h* rather than try to search for an optimal one, and we can find a confidence interval for ${\hat{f}}_{h}^{\prime }(t)$ at every point in scale space and determine whether there is a significant difference from zero at that point.

So for each point (*t*, *h*) in scale space, we wish to test the hypothesis

(4) $$H_{0}:f_{h}^{\prime }\left(t\right)=0\;\text{against}\;H_{1}:f_{h}^{\prime }\left(t\right)\neq 0$$

based on the unbiased estimator ${\hat{f}}_{h}^{\prime }(t)$ of *f*_*h*_′(*t*). Hence, we compute a confidence interval for *f*_*h*_′(*t*),

(5) $${\hat{f}}_{h}^{\prime }\left(t\right)\pm q\hat{\text{SD}}\left[{\hat{f}}_{h}^{\prime }\left(t\right)\right],$$

where *q* is the quantile discussed below, and the observation variance $\;\hat{\mathrm{Var}\;}\;{\hat{f}}_{h}^{\prime }\left(t\right)\left(={\left[\hat{\text{SD}}\left[{\hat{f}}_{h}^{\prime }\left(t\right)\right]\right]}^{2}\right)$ is

(6) $$\hat{\mathrm{Var}\;}\left[{\hat{f}}_{h}^{\prime }\left(t\right)\right]=\frac{1}{N\left(N-1\right)}\overset{N}{\underset{i=1}{\sum }}{\left[K_{h}^{\prime }\left(t-t_{i}\right)-\bar{K_{h}^{\prime }\left(t-t_{i}\right)}\right]}^{2}$$

with *N* the number of samples, and $\bar{K_{h}^{\prime }(t)}$ is the sample mean of *K*_*h*_′(*t*).

This treatment is valid if the normal approximation is valid, that is, if ${\hat{f}}_{h}^{\prime }\left(t\right)\sim 𝒩\left(f^{\prime }\left(t\right),\hat{\mathrm{Var}\;}\left[{\hat{f}}_{h}^{\prime }\left(t\right)\right]\right)$ approximately. The effective sample size (ESS) is defined as

(7) $$\text{ESS}\left(t,h\right)=\frac{\sum_{i}K_{h}\left(t-t_{i}\right)}{K_{h}\left(0\right)},$$

and the normal approximation is considered valid if and only if ESS(*t*, *h*) ≥ 5. Other regions in scale space are considered as inconclusive, see [4] for more details.

The quantile *q* has to be treated with care to correct for multiple testing [4]. The most straightforward way is to assume *m* independent tests such that the quantile becomes

(8) $$q\left(h\right)=\Phi^{-1}\left[\frac{1+{\left(1-\alpha \right)}^{1/m}}{2}\right],$$

where *α* is the confidence level and Φ(*x*) is the cumulative normal Gaussian distribution. We use *α* = 0.05 throughout. The number *m* = *m*(*h*) of independent tests is approximated by

(9) $$m=\frac{N}{\text{av}{\text{g}}_{t}\text{ESS}\left(t,h\right)}.$$

This approximation can be improved upon using extreme value theory as described in [15], which is employed for the analysis in the current paper. Other ways to correct for multiple testing exist and have been used in the literature, such as bootstrapping [4] or false discovery rate (FDR) [16], but results are typically very similar to those found by estimating the number of independent test, and for computational simplicity we use this technique in the following.

Thus, if the confidence interval we arrive at contains zero, we conclude no significant gradient and otherwise label the point as significant positive or negative gradient accordingly. The data are typically presented as a family plot with smooths for a variety of bandwidths together with a SiZer plot that denotes each point in scale space according to the result of the hypothesis test, or a different designation for those regions where the effective sample size is too small.

Here we have considered the case where we have a set of observations at times *t*_*i*_ and wish to estimate an underlying distribution *f*(*t*). The same methodology can be applied to regression problems, as in Section 4. In this case, there is a set of observations *y*_*i*_ at a given time *t*_*i*_, and we wish to find any significant structure in how these change in time. Nonparametric regression involves using a kernel with bandwidth (or scale) *h* and compute an estimate of the curve at a given time *t* using only those data points that are close to *t*, where closeness is defined by the kernel *K*_*h*_(*t*). We use a local linear smoother

(10) $${\hat{f}}_{h}\left(t\right)=\underset{b}{\arg\;\min\;}\overset{N}{\underset{i=1}{\sum }}{\left[y_{i}-\left(a+b\left(t_{i}-t\right)\right)\right]}^{2}K_{h}\left(t_{i}-t\right)$$

as an estimator for the conditional regression function *f*(*t*) = E(*y*_*i*_ | *t*_*i*_ = *t*). Using the derivative ${\hat{f}}_{h}^{\prime }(t)$, the same framework as for the histogram smoother can be applied to construct a scale space map of the significance in a time series. In both cases the resulting significance maps are known as SiZer maps, but we will be careful to point out when we are using density estimation or kernel regression.

## 3. Aggregated Data

**Figure 1:**
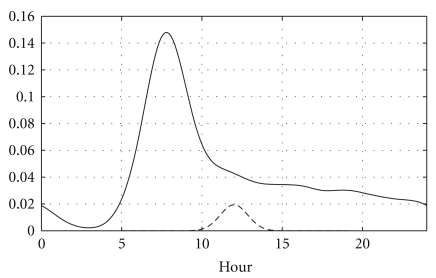
The solid line shows the density estimate *T*_*h*_(*t*), with data from the full cohort, and shows how readings are spread through the day. We have used a Gaussian kernel smooth with the data-driven bandwidth *h* = 51 min. The dashed line shows the kernel scaled for visibility. All data are treated as circular statistics.

Taking all patients into account, we analyzed how often they recorded insulin, at which time of the day and overall trends. In this section we focus only on the time at which recordings were done, and not the actual BGC value.  Figure 1 shows how all BGC readings made by the patients are distributed over 24 hours. This is smoothed by a Gaussian kernel with bandwidth as recorded by the data-driven bandwidth returned by the Sheather-Jones algorithm [11] resulting in a bandwidth of 51 minutes. We have assumed circular statistics to avoid edge discontinuities at midnight, though using a Gaussian distribution rather than the more apt but complicated von Mises distribution since the bandwidth is much smaller than the period, such that the von Mises and Gaussian distribution would be virtually identical. That is, given readings at times *t*_*i*_, *i* = 1 ⋯ *N*, the smooth is

(11) $$T_{h}\left(t\right)=\underset{K\to \infty }{\lim\;}C_{K}\overset{K}{\underset{k=-K}{\sum }}\;\overset{N}{\underset{i=1}{\sum }}K_{h}\left(t-t_{i}+kP\right),$$

where *P* = 24 h, and the normalizing constant is

(12) $$\begin{array}{c} C_{K}=2{\left\{\overset{K}{\underset{k=-K}{\sum }}\;\overset{N}{\underset{i=1}{\sum }}\left[\mathrm{erf}\;\;\left(\frac{\left(k+1\right)P-t_{i}}{h\sqrt{2}}\right)-\mathrm{erf}\;\;\left(\frac{kP-t_{i}}{h\sqrt{2}}\right)\right]\right\}}^{-1},\\ \mathrm{erf}\;\;\left(x\right)=2\pi^{-1/2}\overset{x}{\underset{0}{\int }}\exp\;\;\left(-\mu^{2}\right)d\mu \end{array}$$

is the standard error function. This accounts for edge effects and ensures a continuous distribution across midnight. Since the relevant bandwidths are, by construction of the problem, much less than the period, *h* ≪ *P*, the contribution from the terms with *K* > 1 will be negligible. Thus, we use *K* = 1, such that the sum over *k* extends only to the neighboring nodes (*k* = −1,0, 1) and consider only 0 < *t* < 24 h. There is a significant peak at around 7:45 AM, with a quick drop-off, and thereafter a steady decline over the course of the day. This reflects that most patients take one BGC reading in the morning as per medical recommendation. A few measurements are done over the course of the day with a steady decline in numbers until midnight.

**Figure 2:**
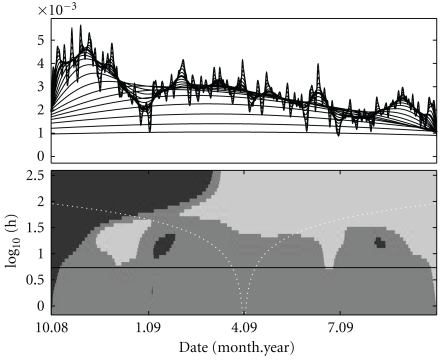
Top: a family plot of the density estimate for the times of BGC readings for the full cohort over the full project period. Each line is a Gaussian kernel smooth with increasing bandwidth. The thick line corresponds to the data-driven bandwidth, *h* = 8.7 days. Bottom: a SiZer plot of the data in the upper figure. In shades from light to dark these correspond to (i) significant negative gradient, (ii) no significant gradient, (iii) not enough data for inference (does not appear in this particular map), and (iv) significant positive gradient. The horizontal line corresponds to the data-driven bandwidth which is displayed in bold in the upper panel. The dashed white lines visualize four times the bandwidth at each scale.

In the upper half of Figure 2 we show a family plot of all readings through the entire trial period using a kernel density estimator with Gaussian kernel smooth and different bandwidths *h*. We choose the range of bandwidths such that we capture all relevant scales, that is, from the smallest scale at which there is a significance found, to the full range of the data. Obviously, at the largest end most features of the data set are smoothed away, while the smallest bandwidths show spurious features that are unlikely to be supported by the data set. Nevertheless, ignoring the falloffs at the ends, some important features emerge. There is a big decline in readings at Christmas time, a period where regular routines tend to be changed. The same effect appears, though with less prominency in summer. The early abrupt increase is due to the start of the study, and after this there is a steady decline in readings. This can be attributed to the effect that the patients find new technology and gadgets exciting and use it more frequently initially, while tiring after some period.

Exploring features that emerge on different scales like these is ideal for the SiZer methodology. Exploring the scale space with time and scale the relevant axes, each point is labelled according to (i) significant negative gradient, (ii) no significant gradient, (iii) not enough data for inference, and (iv) significant positive gradient. The complete map, known as the SiZer map, is shown in the bottom half of Figure 2.

The SiZer plot shows the overall negative trend after an initial steep positive trend, and the two decreases in frequency are also visible as a light area followed by a dark area. These appear at different scales, and a scale space approach is the only way to detect such instances coherently.

## 4. Blood Glucose Concentration Data

**Figure 3:**
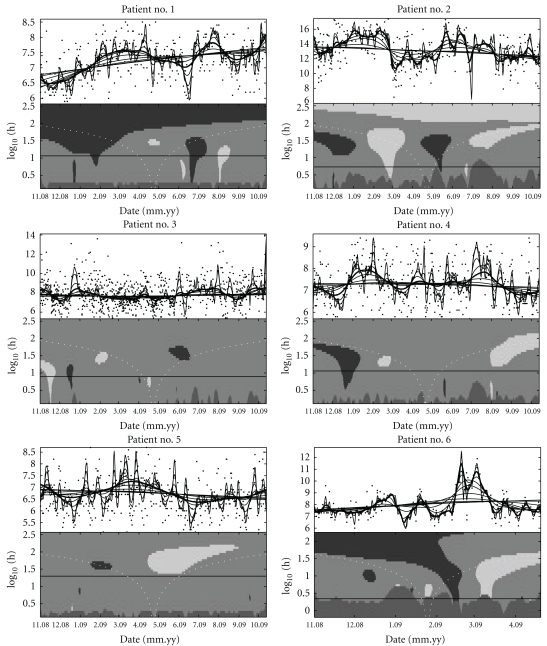
The family smooth and SiZer plots of the kernel regression for BGC values in six patients who recorded their BGC reliably from 1 November 2008 through 13 October 2009, except for patient no. 6, who recorded up to 20 April 2009.

In this section we switch to analysis of the BGC values attained by the individual patients, and in the remainder of the paper, we focus on a subset of the patients. Only patients that have recorded BGC at least once a day for 250 days in total and at least one period of at least 30 days of consecutive days with uninterrupted recordings are considered. This will ensure to include that only patients that have recorded BGC over a significant period of time are included in the further analysis and leaves five patients in the analysis. All these patients have recorded BGC in the period 1 November 2008 through 13 October 2009, which we will use for these patients. Additionally, we include one patient that did not record BGC for the last part of the period, but still has some interesting structure in the recordings. Considering each patient's recording of BGC at a specific time, we get a time series of the BGC values. A SiZer plot for each of these patient's BGC values as a regression analysis is shown in Figure 3.

There are a number of features emerging in the SiZer plots that are not immediately apparent from the BGC readings. Patients no. 1 and no. 6 have an upward trend on the largest scales, patient no. 2 has a significant downward trend on the largest scales, while all other patients have no trend on this scale. It is important to note that many features that appear as prominent in the family plots are not significant, while truly significant features can be obscured in the data. Patient no. 6 is a special case, who did not record BGC reliably after 20 April 2009, but has a clear upwards spike in late February to early March. This patient reported an influenza infection in this period, which is known to raise BGC in many patients, something that is clearly identifiable in this singular case. Also, all patients except no. 5 has a significant increase in BGC in late December, though there is not always a corresponding significant decrease back to a normal level. It is likely that this reflects the change in diet in this period, confounded by a relative sparsity in the recordings. 

An interesting question is whether there is any correlation between the reliability of the recordings for a patient and their BGC readings. However, analyzing the twelve patients independently, and collectively, we find little evidence for such a correlation. To this end, we have visually and quantitatively investigated relations between average intervals between readings in a period and if there is a deviation from the usual pattern of BGC values in that period.

## 5. Periodograms

The same ideas as above can be used in the frequency space to detect significant periodicities at different scales [6]. However, such techniques have only been developed for evenly sampled data, whereas this is clearly not the case here. Nevertheless, we can make a Lomb-Scargle periodogram [17] and do inference on this, though not being a truly scale space idea. For a time series *x*_*j*_ = *x*(*t*_*j*_) the Lomb-Scargle periodogram is

(13) $$\begin{array}{cc} P\left(\omega \right) & =\frac{1}{2{\hat{\sigma }}^{2}}\{\frac{{\left[\sum_{j}\left(x_{j}-\bar{x}\right)\cos\;\;\omega \left(t_{j}-\tau \right)\right]}^{2}}{\sum_{j}{\cos\;}^{2}\omega \left(t_{j}-\tau \right)}\\ & \;\;\;\;+\frac{{\left[\sum_{j}\left(x_{j}-\bar{x}\right)\sin\;\omega \left(t_{j}-\tau \right)\right]}^{2}}{\sum_{j}\sin^{2}\omega \left(t_{j}-\tau \right)}\}, \end{array}$$

where

(14) $$\tan\;\left(2\omega \tau \right)=\frac{\sum_{j}\sin\;2\omega t_{j}}{\sum_{j}\cos\;\;2\omega t_{j}},$$

**Figure 4:**
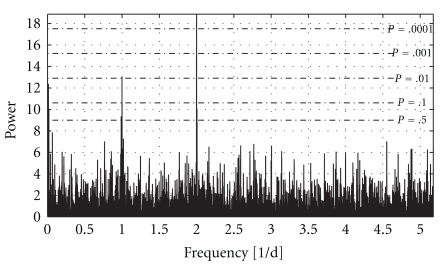
Lomb-Scargle periodogram for patient no. 3 in Figure 3. The significance levels shown are approximate. This patient has three significant peaks in the periodogram with periods 1/*f* equal to 85 days, one day, and 12 hours and respective significance levels *P* = .018, *P* = .0088, and *P* = 2.6 · 10^−5^. The periodogram is plotted up to four times the average Nyquist frequency and with an oversampling factor of five.

$\bar{x}$ and ${\hat{\sigma }}^{2}$ are the sample mean and variance of *x*_*j*_, respectively, and all sums in (13)-(14) run over all *j* = 1,…, *N*. The shift parameter *τ* is introduced as to ensure that the periodogram is independent of an offset in time. This technique is equivalent to a least squares fit to a sinusoidal *α*cos  *ωt* + *β*sin *ωt*. The patient with the most recordings in our dataset is patient no. 3 in the last section, who recorded BGC on 390 days, and a total of 1014 recordings. The Lomb-Scargle periodogram for this patient is shown in Figure 4. The significance of a peak in the periodogram can be approximated by [18]

(15) $$\text{prob}\left(P\left(\omega \right)>z\right)\approx Me^{-z},$$

where *M* is the effective sample size, oversampling taken into account. The significance levels in Figure 4 have been confirmed by Monte Carlo analysis. This patient has three significant peaks, where the least significant is at 85 days, most significant at 12 hours, and one intermediate at 24 hours. The latter is most easily explained as an obvious cycle over the day.

The same patterns emerge for other patients, with one long-term component and the 24 h component. Most patients also have the 12 h component, though with some patients this does not emerge. Note that the spectrogram goes over much larger frequency scales than the Nyquist frequency 1/2Δ, Δ being an average interval between readings. This is a feature of uneven sampling that allows unaliased spectrogram at high frequencies. The long period/low frequency component likely emerges due to positive autocorrelation at small time intervals.

## 6. Conclusions and Future Work

 We have shown how scale space methods developed over the last decade together with information in frequency space can be used to explore features in BGC variations of diabetes patients. These techniques answer in a quantitative way which features of a dataset that really exist in a statistically meaningful way, even if the emergent features appear only on a particular scale. The technique avoids the question of bandwidth selection by investigating all scales simultaneously. The techniques can be valuable even for patients and practitioners to evaluate the effect of changes in lifestyle, medication, or medical aspects of the patient. As such, many of the significant features that are explored in this paper remain unexplained because there is no access to the medical history of the patients, and any conclusions on this are tentative at best.

The work with the mobile platform will continue further and will be deployed among type 1 diabetes patients. These have a more severe condition and record their BGC more frequently and treats the disease with generally greater vigilance. As such, dataset from these patients is likely to contain much more information on their situation, and be useful for doing a predictive analysis that can be employed by the patients as a way to quantify their disease and ease management.

Within a Bayesian framework, one can assume a prior on the BGC value of a patient, with individually adjustable parameters, and then based on information on expected food intake, exercise, and other confounding variables make a posterior distribution on the patient's BGC. The sampling can be made by Gaussian Markov Random Fields [19], Markov Chain Monte Carlo, or other relevant techniques.
